# A Coarse-to-Fine Method for Estimating the Axis Pose Based on 3D Point Clouds in Robotic Cylindrical Shaft-in-Hole Assembly

**DOI:** 10.3390/s21124064

**Published:** 2021-06-12

**Authors:** Can Li, Ping Chen, Xin Xu, Xinyu Wang, Aijun Yin

**Affiliations:** 1 College of Mechanical and Vehicle Engineering, Chongqing University, Chongqing 400044, China; canli@cqu.edu.cn (C.L.); Bachelor_x@163.com (X.X.); xinyu.wang@cqu.edu.cn (X.W.); aijun.yin@cqu.edu.cn (A.Y.); 2 State Key Laboratory of Mechanical Transmissions, Chongqing University, Chongqing 400044, China

**Keywords:** pose estimation, 3D point clouds, shaft-in-hole assembly, robot vision, admittance control

## Abstract

In this work, we propose a novel coarse-to-fine method for object pose estimation coupled with admittance control to promote robotic shaft-in-hole assembly. Considering that traditional approaches to locate the hole by force sensing are time-consuming, we employ 3D vision to estimate the axis pose of the hole. Thus, robots can locate the target hole in both position and orientation and enable the shaft to move into the hole along the axis orientation. In our method, first, the raw point cloud of a hole is processed to acquire the keypoints. Then, a coarse axis is extracted according to the geometric constraints between the surface normals and axis. Lastly, axis refinement is performed on the coarse axis to achieve higher precision. Practical experiments verified the effectiveness of the axis pose estimation. The assembly strategy composed of axis pose estimation and admittance control was effectively applied to the robotic shaft-in-hole assembly.

## 1. Introduction

**Figure 1:**
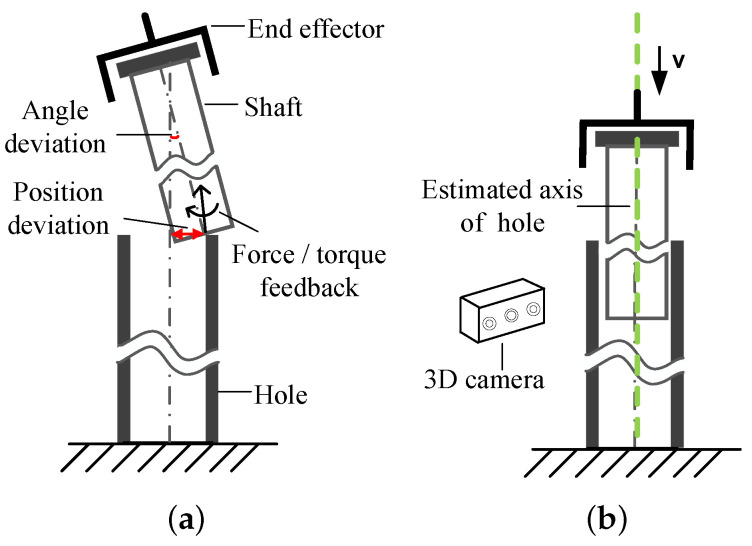
(**a**) Traditional methods prefer to locate the hole by force-guide strategies, where the angle and position deviation between the shaft and the hole are gradually minimized according to F/T feedback. (**b**) In our method by 3D vision, the hole pose is reduced to its axis pose, which is measured by vision in a one-shot. The shaft can be guided into the hole along the axis orientation so that some redundant force control processes are eliminated.

Robotic assembly has attracted many researchers from the fields of automotive, aerospace, and electronics in past years [1,2,3,4,5]. Particularly, in shaft-in-hole assembly, challenges are introduced from the location of the target hole and the smooth insertion of shaft-in-hole. Concerning the location of targets, previous methods relying on pure tactile/force sense [6,7] or hybrid vision-tactile sense [8] can work successfully. Typically, as illustrated in Figure 1a, with the rough pre-positioning of the hole, robots employ force/torque (F/T) sensors to further locate holes exactly to insert the shaft.

However, it can be inferred that the force-guided location for the hole increases the system operation time, which motivates us to promote the accuracy of positioning of the hole by vision sensing before insertion. Furthermore, compared to 2D vision, 3D-vision technologies bring more possibilities to object pose estimation. This is mainly because 3D spatial information of the object can be acquired by a 3D camera in a one-shot while a one monocular camera can only capture two-dimensional images. Using the strategies for 3D vision, both the spatial position and orientation of the target can be obtained. Regarding the insertion of shaft-in-hole, jamming can occur even if the position/orientation error of the object hole by vision measurement is quite small. This is due to the imperfect position control of industrial robots.

Thus, the smooth insertion strategies for shaft-in-hole should be investigated. In general, although force control is inevitable during the insertion process, especially considering the position control error, knowledge of the reliable pose by vision needs to be further explored, which may shorten the time of force control and enable the assembly to be completed faster and more safely. Consequently, we decompose the shaft-in-hole assembly strategy into (i) the pose estimation for hole parts by 3D vision and (ii) the smooth insertion of the shaft into the hole, respectively.

Regarding the object pose estimation by 3D vision, in the past twenty years, a considerable amount of works based on 3D point cloud technologies [9,10,11] have been proposed. Point cloud registration, e.g., the Iterative Closest Point (ICP) algorithm, aligns the point clouds to compute the rigid pose transformation. Despite its simplicity and efficiency, ICP requires a good initialization and is easily trapped in the local optimum [12]. Previous efforts have been made to improve ICP for the global optimal solution [13,14,15]. Nevertheless, templates of point clouds of objects are inevitable to be established in object pose estimation, which causes inconvenience.

In addition to the point cloud registration, some researchers detect objects and acquire their poses by matching models [16,17]. Recently, researchers have begun to apply deep neural networks to process 3D point clouds [18]. Researchers [19] presented SegICP, a novel integrated solution to object recognition and pose estimation, which achieved a 1-cm position error and <5${}^{\circ }$ angle error. Wang et al. [20] developed the 3D-ConvNets to process voxelized input to generate a 3D bounding box and to estimate the target 6D pose.

Generally, deep learning methods are applicable for robotic grasping but not for assembly engineering due to the low precision of pose estimation. Particularly, for the estimation of cylinder-shaped objects, there exist many approaches for cylinder fitting or cylinder detection. These methods can be roughly classified into four categories: (i) random sample consensus (RANSAC) [21], (ii) Hough transform [22], (iii) least squares, and (iv) clustering [23]. Most RANSAC-based methods, e.g., [24,25], directly estimate the cylinder parameters using the information of the point normal. The Hough transform determines cylinders by voting in the parametric space [26].

Least-squares methods fit cylinders by minimizing the distances of points to the cylindrical surface [27,28]. Clustering algorithms extract the cylinder from the raw point clouds based on the Euclidean distances of points and color information. Tran et al. [29] proposed an iterative algorithm for extracting cylinders and estimating their parameters. Kawashima et al. [30] fitted the cylinder surface to terrestrial laser-scanned point clouds using normal-based region growing. Recently, Nurunnabi et al. [31] developed a method for robust cylinder fitting, where the problem was decomposed to circle fitting, orientation, and length determination. In general, these types of methods are practical for fitting cylinders from point clouds.

Concerning the smooth insertion of the shaft into the hole, preventing the shaft from being stuck in the hole during the insertion process is important. However, considering that the position control of robots can not be totally eliminated, jamming will certainly happen if the fit clearance between the hole and shaft is too small. This situation can be solved by admittance control, also known as impedance control [32,33], which is an approach to the control of the dynamical interaction relationship between an end-effector and its environment. With admittance control, robots can adaptively adjust the pose of the end effector toward the hole bottom during the insertion process of shaft-in-hole. Zhao et al. [8] showed the successful implementation of admittance control in peg-in-hole assembly.

As a result, as shown in Figure 1b, the hole pose is simplified to its axis pose consisting of the position and orientation, for which an estimation method is innovatively proposed. Compared to the existing approaches to fitting cylinders, our method focuses on estimating the axis pose in shaft-in-hole assembly, instead of fitting cylinders from cluttered point clouds.

This method provides a reliable axis pose by 3D vision, enabling robots to guide the shaft along the axis into the hole. Meanwhile, admittance control is employed to guaranteed smooth insertion to achieve the shaft-in-hole assembly. More importantly, if one of the assembly targets is the hole with a large aspect ratio (ratio of height to diameter), the accuracy of the axis pose should be guaranteed. Otherwise, a tiny error of axis orientation may imply a large error of position on the axis end, preventing the shaft from entering the hole along the axis.

To the best of our knowledge, although some researchers have solved the peg-in-hole assembly [6,7,8], which is similar to the shaft-in-hole one, their methods are more dependent on force sensing than vision, limiting the efficiency of assembly. As shown in Figure 1, intuitively, it is time-consuming to adjust the shaft pose relative to the hole according to the contact force feedback. In turn, in having a reliable axis pose through vision, robots can reduce the force-guided location operation and may even insert the shaft into the hole in a one-shot. Therefore, aiming to promote the efficiency of shaft-in-hole assembly, we advance the prior works by presenting a novel method for estimating the axis pose. The contributions of our work can be summarized as follows:

(i) Reducing the hole pose to its axis pose, we propose a novel coarse-to-fine method for estimating the axis pose based on 3D point clouds. Our method can provide the pose estimation for the hole, which is reliable for both position and orientation and reaches better performance compared to the popular methods for estimating the axis.
(ii) Under our devised robotic platform, we employ the above method for estimating the axis pose, together with an approach of admittance control, to promote the cylindrical shaft-in-hole assembly. Using this assembly strategy, the shaft-in-hole assembly with a large aspect ratio is accomplished efficiently.

The remainder of this paper is organized as follows: Section 2 presents the robotic platform and principles for assembly. Section 3 describes the coarse-to-fine method for estimating the axis pose. Section 4 and Section 5 provide the experiment and the conclusions of paper, respectively.

## 2. Robotic Platform and Principles for Assembly

### 2.1. Robotic Platform

**Figure 2:**
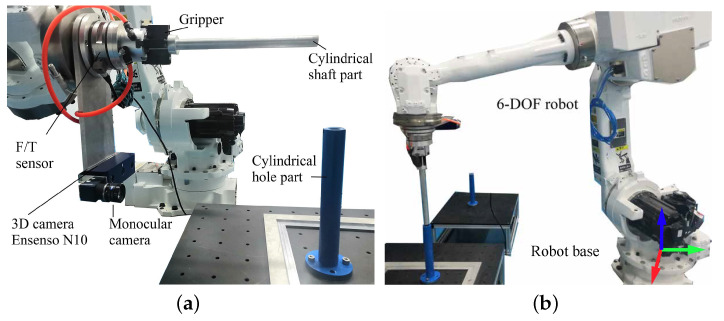
The robotic platform for cylindrical shaft-in-hole assembly. (**a**) The end effector and the equipment mounted on it. (**b**) The six-DOF robot.

Figure 2 shows the robotic platform used to accomplish the cylindrical shaft-in-hole assembly. The six-DOF robot is equipped with a monocular camera, a 3D camera, an F/T sensor, and a gripper on the end effector. In particular, the robot is YASKAWA MOTOMAN-MCL50, whose accuracy of position control reaches ±0.07 mm. The monocular camera is used to locate the cylindrical hole part roughly in 2D, and the 3D camera is for precise pose estimation. The F/T sensor provides force-torque feedback during the insertion of shaft-in-hole.

**Figure 3:**
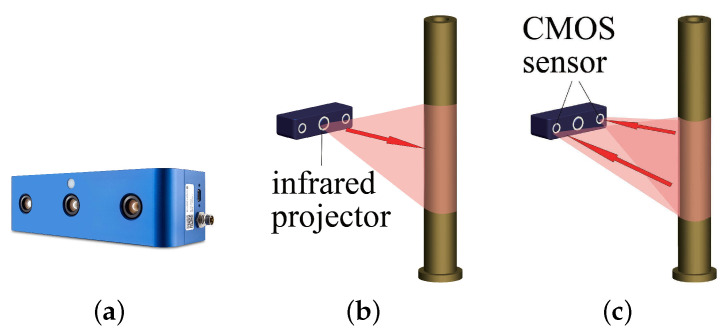
Working principles of a 3D camera: (**a**) The 3D camera Ensenso N10. (**b**) The infrared light is projected onto the surface of a target. (**c**) The infrared spots are collected by CMOS sensors.

The 3D camera is Ensneso N10, and its working principle is presented in Figure 3. The first step is to emit non-visible infrared light spots onto the surface of a measured object by the projector, and then the spots will be collected by two CMOS (Complementary Metal Oxide Semiconductor) sensors. Afterward, the spatial position of each spot can then be calculated by the transformation of coordinates determined by the previous calibration. The final output is the partial surface point cloud of the cylindrical part, which is denoted as a point set $P=\left\{p_{1},p_{2},\ldots ,p_{n}\right\},p_{i}\in R^{3}$. Note that the accuracy of the point position will reach ±0.1 mm if the camera working range is within 300 mm.

### 2.2. Principles for Assembly

**Figure 4:**
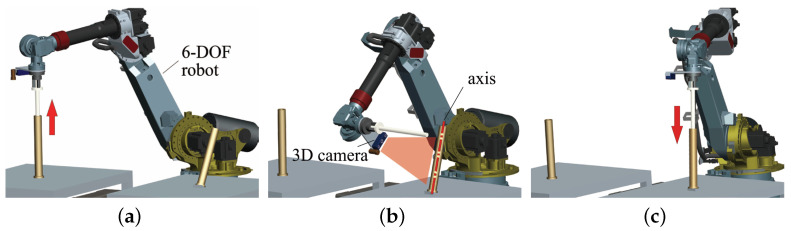
Cylindrical shaft-in-hole assembly. (**a**) The grasp of a shaft. (**c**) The axis pose estimation for the hole. (**c**) The smooth admittance of the shaft into the hole.

Figure 4 presents the assembly process consisting of the grasp of a shaft, the axis pose estimation for a hole, and the admittance of shaft-in-hole. More specifically, first, a cylindrical shaft part is grasped out of a hole by the end effector. Then, another hole is located roughly by the monocular camera. The axis pose of the hole is measured by the 3D camera in a one-shot. After that, with the given axis pose, the end effector is moved to a pre-inserting position on the top of the hole. According to the axis orientation, the shaft can be inserted into the hole. Meanwhile, the admittance control strategy will work to guarantee smooth insertion, preventing the shaft from being stuck in the hole. When the shaft is almost completely in the hole, the robots will release the shaft. Note that the hole parts are fixed on the known tables.

More specifically, for the axis pose estimation, a vector X = $\left[\begin{array}{cc} \rho & \varphi \end{array}\right]$ is used to quantitatively describe the axis pose, in which ρ = ${\left[\begin{array}{ccc} x & y & z \end{array}\right]}^{T}$∈$R^{3}$ is the position of a point on the axis, and φ = ${\left[\begin{array}{ccc} u & v & w \end{array}\right]}^{T}$∈$R^{3}$ is a unit direction vector representing the axis orientation. Regarding the insertion process, due to the error of the axis pose and robot position control, the shaft would inevitably contact the internal surface when it reaches a certain depth, leading to the jamming situation. This situation could be solved with the help of admittance control. During the insertion process, if the shaft interacts with the hole (a linear second-order relationship [33]) the equation

(1) $$M_{d}\ddot{e}+D_{d}\dot{e}+K_{d}e=F_{\mathrm{ext}},$$

can be established, where $e=\left(x-x_{0}\right)$ is the deviation from a desired equilibrium trajectory $x_{0}$, $F_{\mathrm{ext}}$ is the external force from the environment, the constants $M_{d}$, $D_{d}$, and $K_{d}$ represent inertia, damping, and stiffness, respectively. This dynamic relationship in Equation (1) will be maintained to continuously adjust the pose of the end-effector before the robot completes assembly.

**Figure 5:**
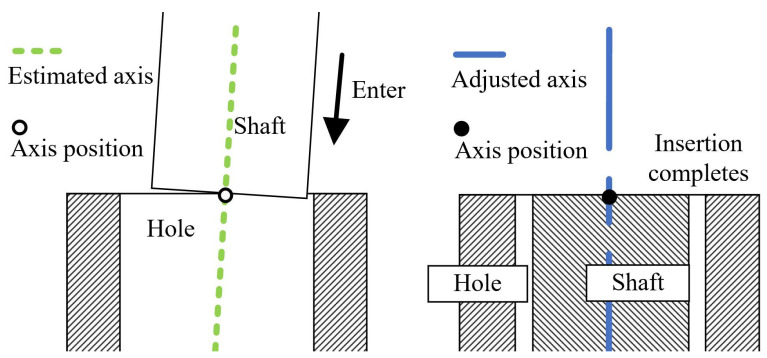
(**Left**) The shaft enters the hole to a certain depth with the estimated axis pose. (**Right**) The adjusted axis pose measured by the end effector.

To sum up, as shown in Figure 5, according to the estimated axis pose of the hole by 3D vision, the shaft grasped by the end effector enters the hole to a certain depth. At the same time, to prevent the shaft from getting stuck in the hole, the admittance control method is applied to adaptively adjust the pose of the end-effector toward the hole bottom until the insertion process is over.

## 3. Method for Estimating the Axis Pose

**Figure 6:**
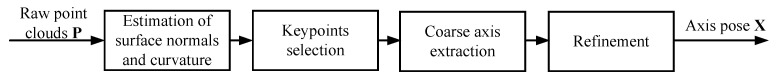
The flow diagram of the proposed coarse-to-fine method for axis pose estimation.

In this section, we illustrate the proposed method for estimating the axis pose in detail. The coarse-to-fine strategy for measuring the axis pose of the hole is given in Figure 6. The procedure of the method mainly includes keypoint selection, coarse axis extraction, and refinement. More specifically, given the raw point cloud P, the normals and the curvature will be first estimated. Then, keypoints are selected according to the curvature. Based on the keypoints, a coarse axis can be calculated. Lastly, the refinement is performed on the coarse axis to achieve higher precision, outputting the resulting axis pose X.

### 3.1. Keypoint Selection by Curvature

**Figure 7:**
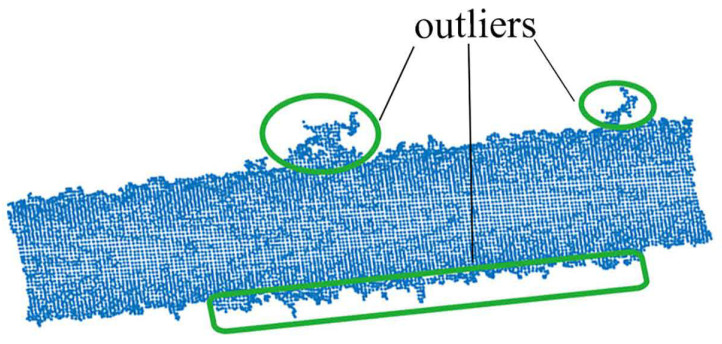
The partial surface point cloud of the cylindrical part.

There always exist the outliers and the noise in the raw point clouds acquired by the 3D camera, as shown in Figure 7, which will inevitably lead to inaccuracy of the pose estimation. To alleviate the influence of the outliers and noise, point fitting the cylindrical surface best should be separated from the raw point cloud. Since the points on the cylindrical surface share the same curvature, the points that meet the given curvature condition will be kept as the keypoints while other points are filtered out.

**Figure 8:**
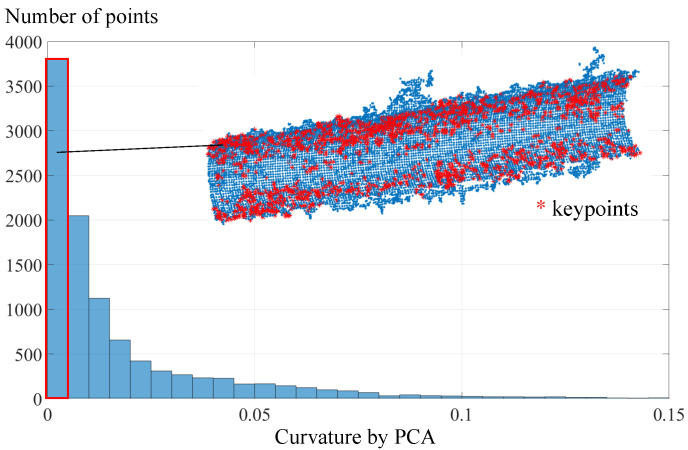
Keypoint selection by curvature.

Principal Component Analysis (PCA) can be used as an approach to approximate the surface variation around a point [34]. Performing PCA on the *k* neighbors set $p^{k}$ of a point $p_{i}$, the positive semi-definite 3 × 3 covariance matrix C is written as

(2) $$C=\frac{1}{k}\sum_{i=1}^{k}\left(p_{i}-\bar{p}\right)\cdot {\left(p_{i}-\bar{p}\right)}^{T},$$

(3) $$C\cdot t_{j}=\lambda_{j}\cdot t_{j},\;j\in \left\{0,1,2\right\},$$

where $p_{i}\in p^{k}$ and $\lambda_{0}<\lambda_{1}<\lambda_{2}.$ The surface variation $\sigma_{p}$ in a neighborhood centered around *p* is estimated by

(4) $$\sigma_{p}=\frac{\lambda_{0}}{\lambda_{0}+\lambda_{1}+\lambda_{2}},$$

where $\lambda_{0}=\min\left\{\lambda_{j}\right\}$. Note that the neighbors set $p^{k}$ is found by a radius search, and the radius is empirically set as 5% of the point cloud span. Figure 8 describes the curvature distribution of the point cloud of a cylindrical hole part. The smoother the point cloud surface is, the smaller the surface variation $\sigma_{p}$. Based on this, the points whose curvature values $\sigma_{p}=0$ are saved, while others are filtered out.

### 3.2. Coarse Axis Extraction Based on the Surface Normals

**Figure 9:**
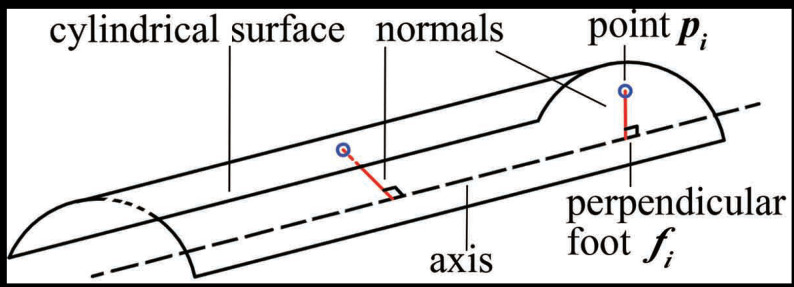
Geometrical constraints between the axis and the surface normals in a cylinder. The axis is perpendicular to and intersects the normals. $p_{i}$ is a point on the surface and $f_{i}$ is a foot point on the axis.

Theoretically, two points with their normals determine a cylindrical axis [25], as shown in Figure 9. This is because the axis is perpendicular to and intersects the normals. As a result, we propose to estimate the pose of an axis based on the point normals.

**Figure 10:**
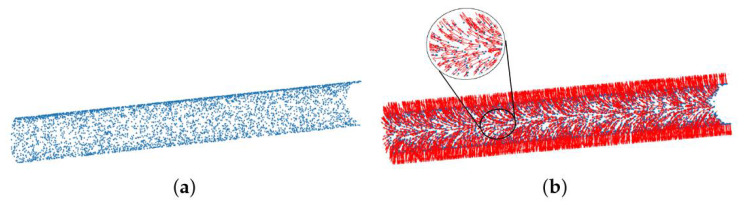
Surface normals estimation: (**a**) The artificial point cloud. (**b**) Surface normals, marked by red arrows, are estimated via PCA.

To briefly explain the fundamentals of the axis extraction method, the artificial point cloud P is adopted as the test object, as shown in Figure 10a. Surface normal estimation can be accomplished via PCA [35]. According to Equations (2) and (3), the eigenvector $t_{0}$ that corresponds to the smallest eigenvalue $\lambda_{0}$ approximates the normal vector $+n=\left\{n_{x},n_{y},n_{z}\right\}$ or −n. Furthermore, the viewpoint $o_{p}$ is usually set to orient all normals $n_{i}$ consistently toward it, and the normals $n_{i}$ have to satisfy the equation

(5) $$n_{i}\cdot \left(o_{p}-p_{i}\right)>0.$$

Subsequently, the combination of the point set P and their normal vectors can be represented by a data matrix V,

(6) $$V=[\begin{array}{ccc} v_{1} & ,\ldots , & v_{n} \end{array}],$$

where $v_{i}={\left[\begin{array}{cccccc} x_{i} & y_{i} & z_{i} & n_{ix} & n_{iy} & n_{iz} \end{array}\right]}^{T}$ is composed of point coordinates and its normal vector. The result of the surface normals estimation is visualized in Figure 10b.

Once estimated, in principle, the axis can be extracted according to Figure 9. However, there are orientation errors in the estimated normal vectors. Despite this, most normal vectors are close to the ground-truth values. Therefore, a straight line supported by most normal vectors is considered as a coarse axis, for which we put forward a RANSAC-based method.

The pseudo-code for coarse axis extraction is shown in Algorithm 1. The input of the algorithm is a set of 3D points and their normal vectors, expressed as Equation (6). The output includes two points $\left\{l^{*},m^{*}\right\}$ determining an axis and a initial radius. Critically, in  step 4, how a potential axis $\left\{l,m\right\}$ is calculated follows Equations (7)–(10).
**Algorithm 1:** Coarse axis extraction.
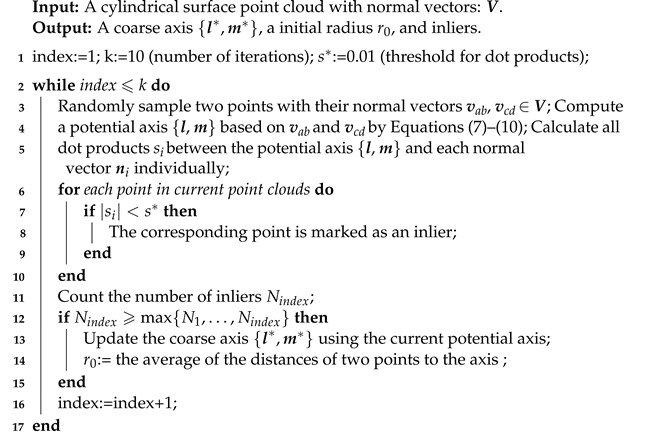


First, a potential axis $\left\{l,m\right\}$ is defined as

(7) $$\frac{x-x_{m}}{x_{l}-x_{m}}=\frac{y-y_{m}}{y_{l}-y_{m}}=\frac{z-z_{m}}{z_{l}-z_{m}},$$

where $l={\left[\begin{array}{ccc} x_{l} & y_{l} & z_{l} \end{array}\right]}^{T}$ and $m={\left[\begin{array}{ccc} x_{m} & y_{m} & z_{m} \end{array}\right]}^{T}$. l and m can be represented as

(8) $$\left[\begin{array}{c} m\\ l \end{array}\right]=\left[\begin{array}{c} k_{1}\left(b-a\right)+a\\ k_{2}\left(d-c\right)+c \end{array}\right],$$

where $n_{\mathrm{ab}}=\left(b-a\right)$, $n_{\mathrm{cd}}=\left(d-c\right)$, a, b, c, and d are the endpoints of normals, and $k_{1}$ and $k_{2}$ are indetermined coefficients to be solved. Due to the perpendicular constraint, the dot product between a potential axis $\left\{l,m\right\}$ and a normal $n_{ab}$ or $n_{cd}$ equals to 0, i.e.,

(9) $$\left[\begin{array}{c} \left(l-m)\cdot (b-a\right)\\ \left(l-m)\cdot (d-c\right) \end{array}\right]=0.$$

Then, we substitute Equation (8) into Equation (9), and $k_{1}$ and $k_{2}$ will be solved. Moreover, Equation (7) can be expressed as

(10) $$\left[\begin{array}{c} x\\ y\\ z \end{array}\right]=\left[\begin{array}{c} u\\ v\\ w \end{array}\right]t+\left[\begin{array}{c} x_{m}\\ y_{m}\\ z_{m} \end{array}\right],$$

where ${\left[\begin{array}{ccc} u & v & w \end{array}\right]}^{T}$ is the unit direction vector and *t* is an coefficient.

After a potantial axis is computed, the dot products between each normal vector and the axis are calculated. The dot product does not always equal 0 due to the normals with errors, and thus a threshold $s^{*}$ is set to judge whether the normal of a point $p_{i}$ is perpendicular to the axis. If a dot product $s_{i}$ satisfies $s_{i}<s^{*}\left(s^{*}=0.01\right)$, then the normal is considered to be perpendicular to the axis, and the corresponding point is classified as an inlier, otherwise it is an outlier and to be removed.

**Figure 11:**
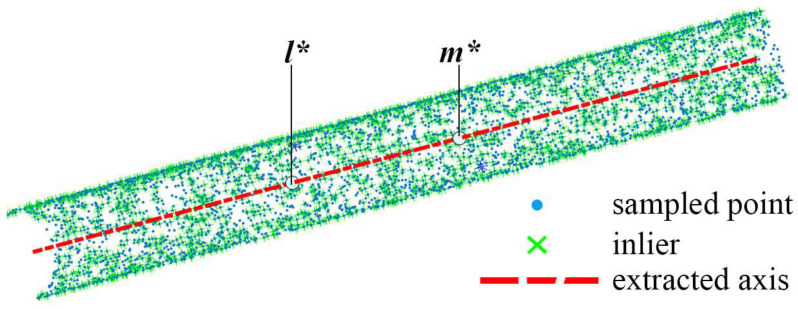
An axis $\left\{l^{*},m^{*}\right\}$ is extracted by Algorithm 1.

On the whole, to obtain the coarse axis, 10 iterations are set to find the axis that is supported by the most inliers. The number of iterations in Algorithm 1 is set according to the strategy of RANSAC [21]. Particularly in each iteration, a potential axis is acquired according to Equations (7)–(10), and then the dot products are calculated for the classification of inliers. After 10 repeats of the procedures, the greatest number $\max\left\{N_{i}\right\}$ is recorded, and the corresponding axis is considered to be the coarse axis. As shown in Figure 11, an approximate axis passing through $l^{*}$ and $m^{*}$ is extracted by Algorithm 1. Although the extracted axis is close to the real one, the algorithm requires further improvement if the point clouds are collected from real scenes.

### 3.3. Refinement of the Coarse Axis by Iterative Robust Least Squares

The axis by Algorithm 1 can only serve as a coarse one due to the limited precision of normals estimation and the errors of point clouds. Consequently, the coarse axis is required to be further refined. The refinement is implemented by iterative robust least squares, which is presented in Algorithm 2. The inputs, including a coarse axis, an initial radius, and the inliers, will be processed to acquire the corresponding refined results.
**Algorithm 2:** Refinement of the coarse axis.
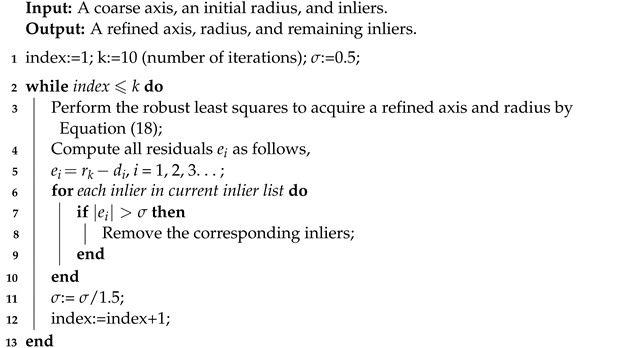


Particularly, in the first iteration, the specific implementation of robust least squares is as follows. The residual $e_{i}$ between the distance $d_{i}$ and the initial radius $r_{0}$ is given by,

(11) $$d_{i}=r_{0}+e_{i},$$

where $d_{i}$ is the distance from a inlier point $p_{i}$ to the coarse axis. $d_{i}$ is computed as below,

(12) $$d_{i}={∥p_{i}-f_{i}∥}_{2},$$

where $f_{i}$ is a foot point on the coarse axis and can be represented by Equation (10) as follows,

(13) $$f_{i}=\left[\begin{array}{c} u\\ v\\ w \end{array}\right]t+\left[\begin{array}{c} x_{m}\\ y_{m}\\ z_{m} \end{array}\right].$$

Meanwhile, $\bar{p_{i}f_{i}}$ is a normal line that is perpendicular to the axis whose direction vector is ${\left[\;u\;v\;w\;\right]}^{T}$, thus one has

(14) $$\left[\begin{array}{ccc} u & v & w \end{array}\right]\cdot \left(\;p_{i}-f_{i}\;\right)=0.$$

Substitute Equation (13) into Equation (14). Then, the solution is,

(15) $$t=-\frac{u\left(x_{m}-{p_{i}}_{x}\right)+v\left(y_{m}-{p_{i}}_{y}\right)+w\left(z_{m}-{p_{i}}_{z}\right)}{u^{2}+v^{2}+w^{2}}.$$

Therefore, let the perpendicular foot $f_{i}$ be $f_{i}$$(x_{m},\;y_{m},\;z_{m},\;u,\;v,\;$$w,\;p_{i})$, then the distance $d_{i}$ can be represented by $d_{i}(x_{m},\;y_{m},\;z_{m},\;u,\;$v,w,$p_{i})$. Afterward, considering all residuals $\sum_{i=1}^{k}e_{i}$, one has the cost function penalizing the distances of the inlier points to the coarse axis, i.e.,

(16) $$J\left(x\right)=\sum_{i=1}^{k}{\left[r_{0}-d_{i}\left(x_{m},\;y_{m},\;z_{m},\;u,\;v,\;w,\;p_{i}\right)\right]}^{2},$$

where $x={\left[\begin{array}{ccccccc} x_{m} & y_{m} & z_{m} & u & v & w & r_{0} \end{array}\right]}^{T}$ is the optimization variable and $p_{i}$ is a given inlier point. Meanwhile, to avoid a refined axis being weakened by few large residuals, a robust kernel is employed, e.g., the Huber kernel [36],

(17) $$H\left(e\right)=\left\{\begin{array}{cc} \frac{1}{2}e^{2} & \;\mathrm{if}\;|e|\leq \delta ,\\ \delta \left(\left|e\right|-\frac{1}{2}\delta \right) & \;\mathrm{otherwise}. \end{array}\right.$$

As a result, one formulates the robust least squares as below,

(18) $$\min_{x}J\left(x\right),$$

where J(x) is the cost function.

In total, 10 iterations are set to obtain the final refined axis, i.e., the outputting axis pose X in Figure 6, which consists in the position and orientation of the axis. During the iterations, some redundant inliers that cause large residuals are filtered out according to line 8 of Algorithm 2. It is worth noting that the number of iterations in Algorithm 2 could be set judging by the convergence of the radius.

## 4. Experiments and Assessment

Section 4.1 describes the procedure of the method for estimating the axis pose. Section 4.2 provides a comparison of our method with others. Section 4.3 verifies the effectiveness of the axis pose estimation in shaft-in-hole assembly. Section 4.4 further analyzes the error of the axis pose. Finally, Section 4.5 discusses the superiority of the proposed assembly strategy.

### 4.1. Procedure of the Axis Pose Estimation

**Figure 12:**
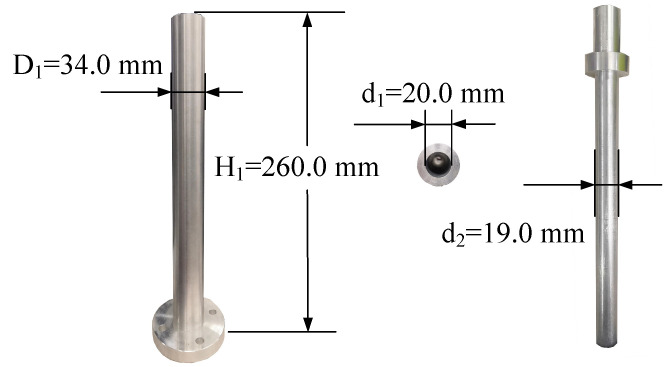
The cylindrical hole and shaft and their basic size.

In this section, we take the cylindrical hole shown in Figure 12 as the test object. In particular, the surface point clouds of the hole are acquired using a 3D camera from four various viewpoints. The axis of the test object is estimated by the coarse-to-fine method of Section 3, which consists of coarse axis extraction and refinement. Despite the various viewpoints of the camera, the resulting radius should be equal to the ground-truth, i.e., $\frac{D_{1}}{2}$. To reflect that the accuracy of the refined axis is higher than that of the coarse one, i.e., the effectiveness of the coarse-to-fine method, the residuals before refinement are compared to those after refinement. Specifically, the residuals are calculated as follows,

(19) $$\begin{array}{c} e_{i}^{in-c}=d_{i}^{in-c}-\frac{D_{1}}{2},\\ e_{i}^{in-r}=d_{i}^{in-r}-\frac{D_{1}}{2}, \end{array}$$

where $d_{i}^{in-c}$ and $d_{i}^{in-r}$ are the distances of the inlier points to the coarse axis and the refined one, respectively.

**Figure 13:**
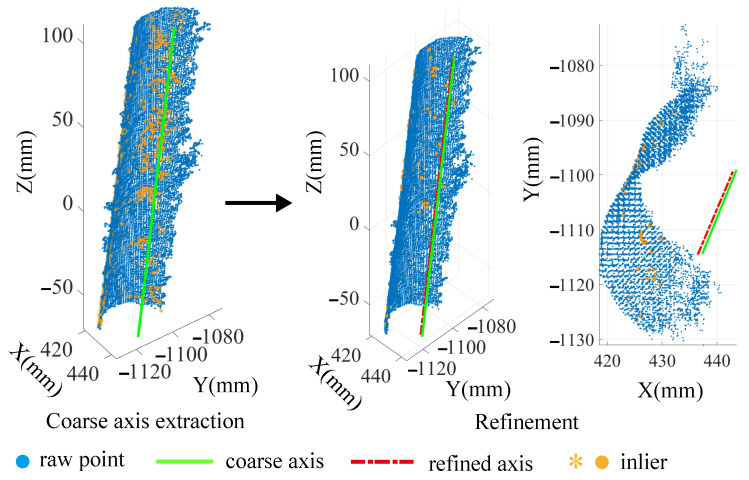
Implementation of the proposed method for estimating the axis.

One of the implementations of the coarse-to-fine method is shown in Figure 13. Specifically, the raw point cloud is first captured from the external surface of a hole by the 3D camera. With the keypoints selected from the raw points, a coarse axis, along with the supporting inliers and the initial radius, is extracted by Algorithm 1. Then, the coarse axis is refined to achieve higher precision by Algorithm 2. Meanwhile, the initial radius is optimized, and the redundant inliers are removed.

**Figure 14:**
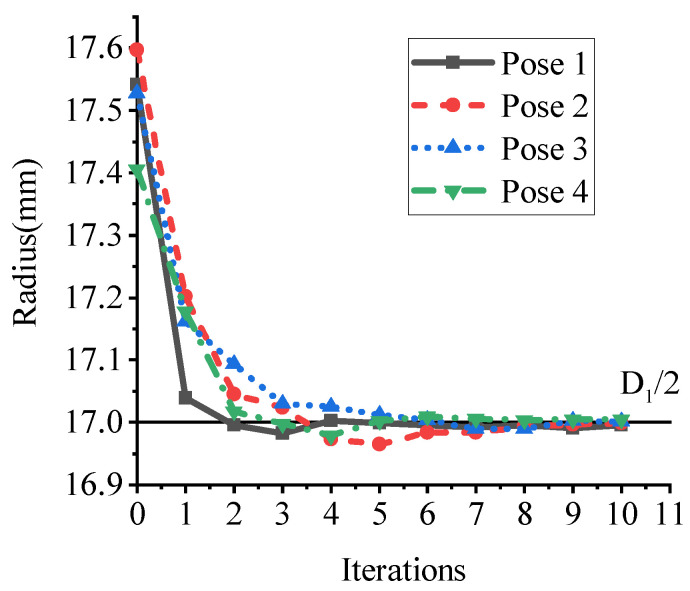
The radius value during the optimization.

Figure 14 provides four various point clouds of the hole and the specific values of the radius during the optimization. The initial radius is obtained after the coarse axis extraction, which can be seen in iteration 0. It shows that a good initial value of radius can be achieved by coarse axis extraction. After 10 iterations, the estimated radius converged to the real radius $D_{1}/2$ by refinement. This illustrates the effectiveness of the coarse-to-fine method.

**Figure 15:**
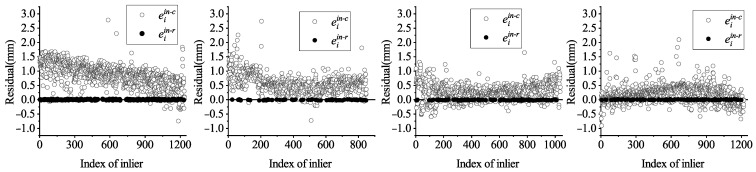
Residuals before and after refinement.

Additionally, the results of the residuals are presented in Figure 15. Before refinement, all $e_{i}^{in-c}$ are scattered around y=0, which implies that a certain error of orientation and position exists in the initial coarse axis. In contrast, after refinement, all $e_{i}^{in-r}$ are evenly distributed on y=0, which illustrates that those errors are almost eliminated. Comparing both of them, it can be inferred that the coarse axis extraction provides a relatively reasonable value of axis, and the accuracy of the coarse axis is indeed improved by refinement. Although there are large residuals before refinement, they do not affect the refined axis due to iterative robust least squares.

To sum up, coarse axis extraction can provide the appropriate initial value, and refinement promotes the accuracy of the coarse axis.

### 4.2. Comparisons between Our Method and Others

**Figure 16:**
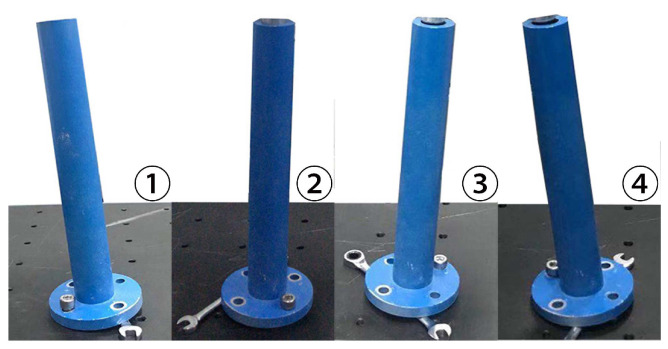
Target hole parts are fixed with four various poses, where differences of both orientation and position among those targets are set to be significant.

To further illustrate the performance of the proposed method for estimating the axis pose, we provide more comparative results. Specifically, the hole is fixed at four different poses, shown in Figure 16, where the differences between any two orientations are large enough and their positions are also different from each other. The intuition behind this setup is to verify whether the methods of pose estimation are reliable enough in handling targets with various poses. The axis poses were estimated by our method, RANSAC [21], PCA [37], and ICP [38]. Among these methods, the RANSAC method estimates the axis in an iterative way as with Algorithm 1.

**Figure 17:**
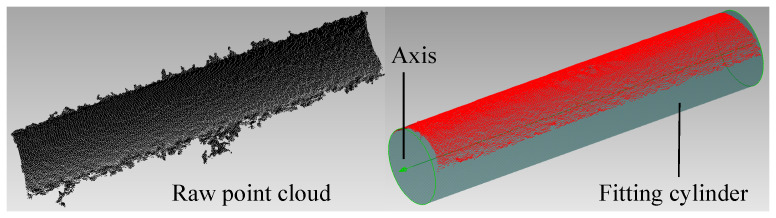
Axis pose determined by the commercial software Geomagic Studio (GS).

The PCA-based method finds the orientation of the axis by calculating the principal direction of the points with normals and locates the position of the axis by fitting the circle. ICP obtains the axis pose by point cloud registration. The axis poses as determined by a well-known commercial software Geomagic Studio (GS) are employed as the comparisons, as shown in Figure 17. In GS, one can manually remove outliers, reduce noise, smooth the point cloud, and perform cylinder fitting to acquire the axis. Thus, the axis by GS has relatively high precision. Additionally, to show the accuracy of the estimated axis on both the orientation and position, the results were compared based on criteria as follows,

(20) $${RMSE}_{\phi }={\left(\frac{1}{n}\sum_{i=1}^{n}\phi_{i}^{2}\right)}^{1/2},$$

(21) $${RMSE}_{d}={\left(\frac{1}{n}\sum_{i=1}^{n}d_{i}^{2}\right)}^{1/2},$$

where $\phi_{i}\left({}^{\circ }\right)$ and $d_{i}$(mm) are the angle and position difference, respectively. The angle difference refers to the angle between two axes, and the position difference is equal to the distance between the axis endpoints. Especially when calculating the position difference, we make the axis endpoints fixed at specific *z* values.

**Figure 18:**
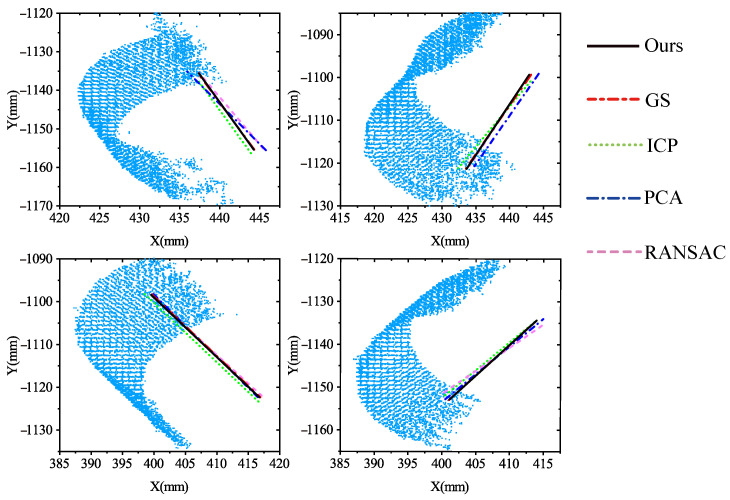
Projection of the estimated axes on the XOY plane by our method, GS, PCA, ICP, and RANSAC.

**Figure 19:**
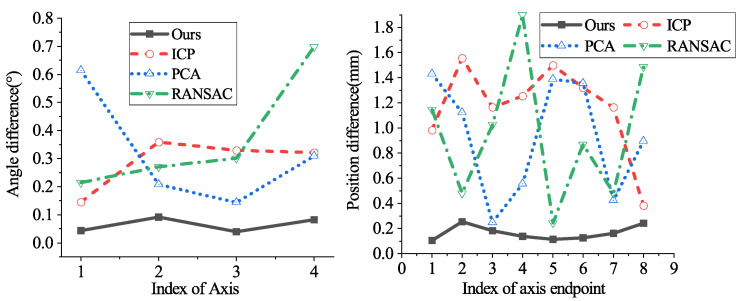
Differences between our method, ICP, PCA, RANSAC, and GS.

Figure 18 provides the projection of the estimated axes on the XOY plane by our method, GS, RANSAC, PCA, and ICP, a total of four groups of experiments. Particularly, the axes by ours and those by GS are almost overlapping. Concerning the comparisons between our method, ICP, PCA, RANSAC, and GS, Figure 19 shows the position differences and angle differences. Note that the position differences refer to the differences between the axis endpoints, which are absolute values. Intuitively, it is shown that the axes by our method are the closest to those by GS regarding both orientation and position, while the axes by other methods have relatively large differences from those by GS.

Table 1 presents the specific value of ${RMSE}_{\phi }$ and ${RMSE}_{d}$. In terms of the axis orientation, RANSAC had the worst performance since the accuracy of the estimated axes was easily corrupted by a few outliers and noise. Although the ICP and PCA algorithm achieved better results than RANSAC, they still did not outperform our method. Regarding the axis position, our method performed best, while others had lower accuracy. This is because we strictly selected keypoints in the smooth area by setting the curvature ($\sigma_{p}=0$) and further saved the inliers for refinement. It is worth noting that the angle difference by our method is so tiny that it will not result in a large deviation on the axis endpoint. As a result, our method is capable of estimating the axis pose with relatively high precision.

### 4.3. Effectiveness of the Axis Pose Estimation in Shaft-in-Hole Assembly

In this section, the method of axis pose estimation, coupled with admittance control, was employed to accomplish the shaft-in-hole assembly, which was our ultimate goal. The shaft and hole with their size information are presented in Figure 12. In each experiment, as shown in Figure 5, we had two various with close axis poses, i.e., the estimated axis pose before insertion and the adjusted one after insertion.

The former was calculated by the coarse-to-fine method, while the latter one was measured by the end effector. Then, the error of the estimated axis pose can be learned by comparison between the estimated axis pose and the adjusted axis pose. Although the adjusted axis measured by the end effector was not perfectly equal to the ground-truth one, it was sufficiently accurate to employ the adjusted axis as the comparison to verify the effectiveness of axis pose estimation in assembly.

**Figure 20:**
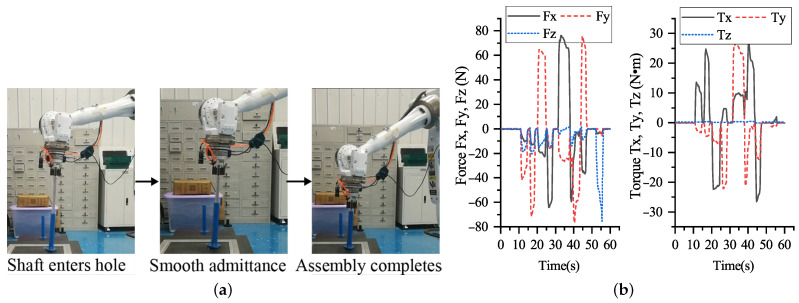
(**a**) Insertion process of shaft-in-hole. Given the estimated axis pose, the shaft moves into the hole along the axis orientation. Then, the shaft continues to move toward the hole bottom with the help of admittance control. (**b**) Force Torque feedback during the insertion for shaft-in-hole.

Figure 20 presents a process of the shaft-in-hole insertion and the corresponding force and torque feedback during the insertion process. In the beginning, the shaft successfully moves into the hole along the axis orientation from a pre-insertion position. Meanwhile, the force and torque are unchanged at nearly zero before the shaft contacts closely the inner surface of the hole. After the shaft reaches a depth, the shaft interacts with the hole, leading to variations of the force and torque.

Judging from the magnitude and orientation of the F/T feedback, the robot adaptively adjusts the pose of the end effector to avoid excessive force and torque. It is worth mentioning that the magnitudes of force and torque are maintained within 80 N and 30 N·m, respectively. As a result, the shaft is not stuck in the hole; instead, it can be completely inserted into the hole, which is so-called smooth admittance. Lastly, the F/T feedback is again near zero when the insertion is completed.

To quantify how much adjustment of the axis pose is made by admittance control, we made a comparison between the estimated axis poses before the insertion and the adjusted axis pose after the insertion as shown in Figure 5. The estimated axis pose is given by our method while the adjusted axis pose is measured by the end effector and displayed on the robot teaching device. More specifically, the angle and position differences between the axes are calculated out.

**Figure 21:**
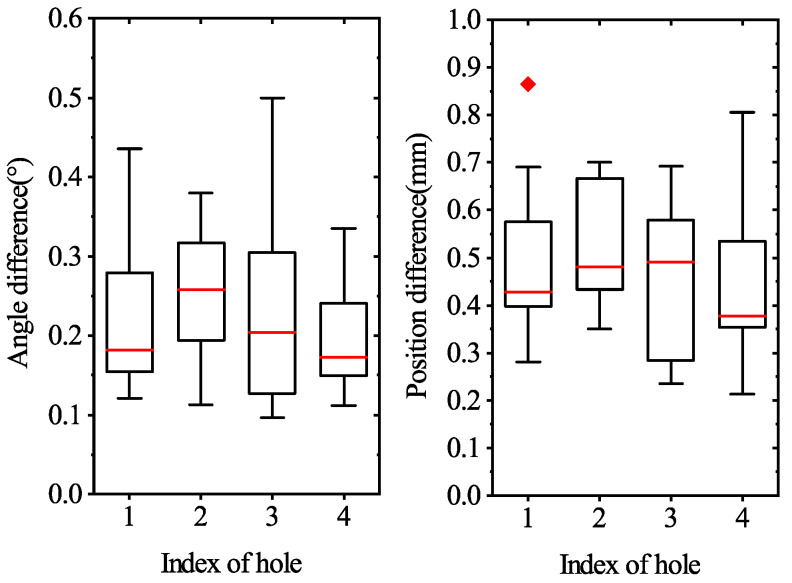
Box plot of the comparison between the estimated axis pose and the adjusted axis pose on the four target holes.

We experimentally conducted 10 successful assemblies for each target hole in Figure 16, respectively, for a total of 40 successful assemblies. Figure 21 provides the comparison between the estimated axis pose and the adjusted axis pose. It shows that all estimated axis poses were adjusted by admittance control, by around 0.15–0.3${}^{\circ }$ on orientation and 0.4–0.6 mm on position. It is worth noting that there was no linear correlation between the adjustment of angle and that of position.

**Figure 22:**
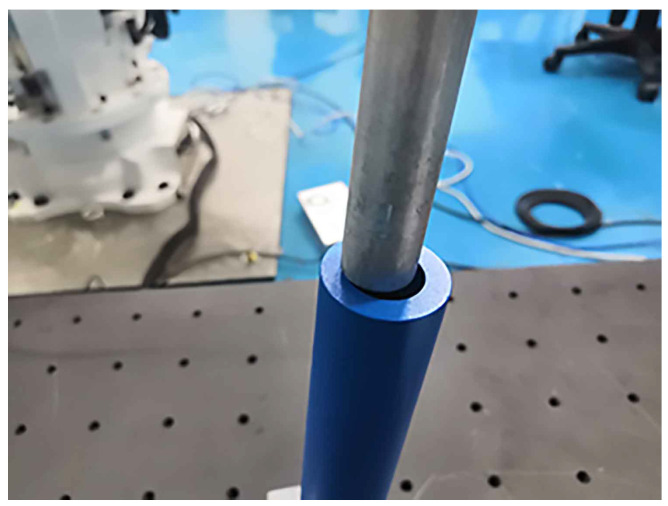
A failure case of assembly, in which the shaft could not even be aligned with the hole.

Additionally, we also conducted several assembly experiments by the pose estimation methods mentioned in Section 4.2, i.e., ICP, PCA, and RANSAC. However, with the rough poses by those approaches, the assembly frequently failed, where the shaft could not even be aligned with the hole, let alone be inserted into the hole, as shown in Figure 22.

To quantify how far those unsatisfactory poses are from the correct ones, we manually moved the end-effector to the correct pose by the robot teaching device and made a comparison between them. For convenience, the results mainly focus on the position difference, as presented in Table 2. The position errors of >5 mm resulting from ICP, PCA, and RANSAC prevented the shaft from entering the hole. Compared to those approaches, the proposed coarse-to-fine method for pose estimation had better performance in assembly.

In summary, according to the axis pose by the proposed method, the shaft can be guided into the hole safely. Moreover, the method coupled with admittance control provides an efficient way to accomplish the shaft-in-hole assembly.

### 4.4. Error Analysis of Axis Pose in Robotic Platform

**Figure 23:**
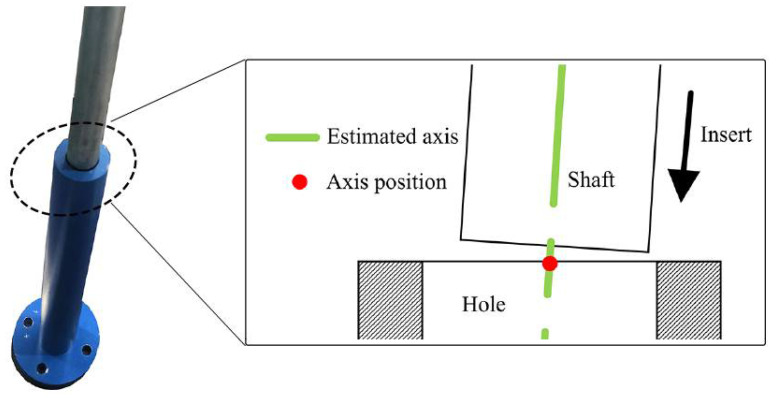
Axis position at the entrance of the hole.

In this section, we further analyze the error of axis pose estimation. Recall that the robot will insert the shaft into the hole along the estimated axis. As shown in Figure 23, at the entrance of the hole, the position of the axis determines whether the shaft can move into the hole along the axis. Therefore, under the robotic platform, we should take into consideration the position errors of the axis at the hole entrance. The knowledge of the error range shows the confidence of the axis pose estimation.

Since the robot platform is calibrated in advance, the systematic error is supposed to be negligible. We assume that the position part of an axis is corrupted as below,

(22) $$\rho =\xi_{\rho }+\bar{\rho },$$

where $\bar{\rho }$ is the mean of ρ, $\xi_{\rho }\in R^{3}$ is zero-mean Gaussian perturbation with covariance matrice $\Sigma_{\rho }$. In particular, since the holes are fixed on the known table, the height of the hole entrance is 120 mm approximately. Then, ρ is set as ${\left[\begin{array}{ccc} x & y & 120 \end{array}\right]}^{T}$, and the covariance matrix $\Sigma_{\rho }$ is 2 × 2.

**Figure 24:**
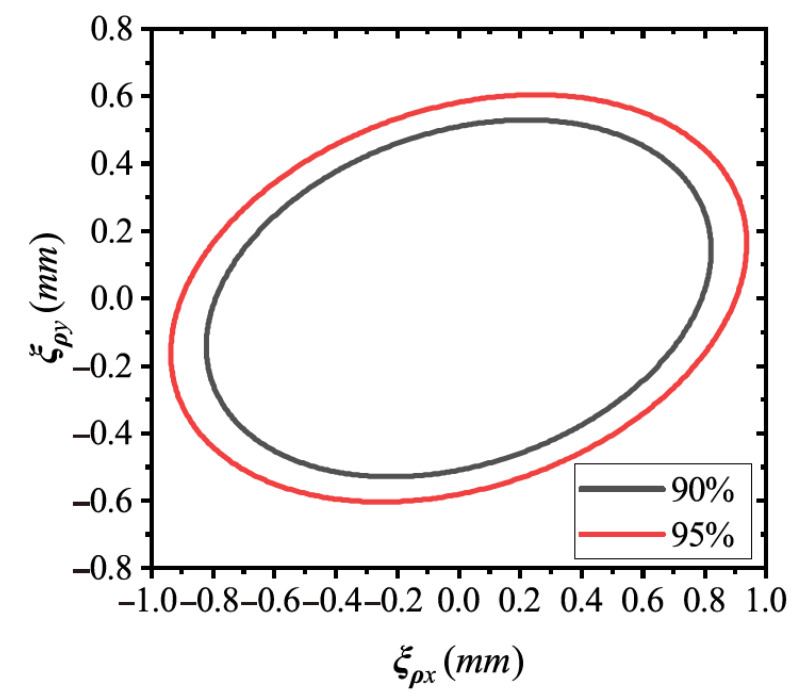
Error ellipses with 90% and 95% confidence.

We experimentally collected 100 point clouds for each target in Figure 16 and estimated the axis poses for a total of 400 point clouds. Especially during the collection, the holes were fixed with four various poses, while the point clouds were captured from different camera viewpoints as much as possible. By statistical methods, we obtained the solutions to $\Sigma_{\rho }$. Figure 24 visualizes the error ellipses by $\Sigma_{\rho }$. The error of the axis position remained at a low level: <0.8 mm with 90% confidence and <1 mm with 95% confidence.

Remember that the accuracy of robot position control reached ±0.07 mm, and the accuracy of point clouds generated by the 3D camera (Ensenso N10) can reach ±0.1 mm. Both the point cloud quality and position control accuracy would impact the performance of the proposed assembly strategy. On the one hand, the error of point clouds can corrupt the quality of the estimated axis pose. Despite this, it would not prevent the shaft from being guided into the hole according to the estimated axis pose, since the error of point clouds is negligible compared to the fit clearance between the shaft and hole (1 ± 0.05 mm).

On the other hand, the position control accuracy influences the insertion of shaft-in-hole assembly. The assembly target is the cylinder part with a large aspect ratio, which means that the length of the insertion path is relatively long. Even though the shaft can be guided into the hole according to the estimated axis pose, the shaft is most likely to be stuck in a certain depth of the hole due to imperfect position control accuracy. However, this situation can be solved by admittance control approaches. In summary, the impacts from position control accuracy and camera quality are within an acceptable level.

### 4.5. Discussion

Concerning the problem of object location in shaft-in-hole assembly, our strategy employs 3D vision to measure the reliable axis pose of the hole in a one-shot, while other force-sensing guided approaches take a great deal of time to locate the hole gradually according to force feedback (see Figure 1). The assembly execution time mainly depends on the robot operation speed, which is manually set. Under the same setup, consequently, the assembly execution time could be shortened by our strategy since some redundant force-sensing tasks for locating holes are replaced by 3D vision-sensing ones.

## 5. Conclusions

Aiming to promote the robotic shaft-in-hole assembly, in this work, we investigated the object pose estimation and proposed a coarse-to-fine method for estimating the axis pose of the hole using 3D vision. Having a reliable axis pose, robots can move the shaft into the hole along the axis orientation without the redundant force-guide location for holes. Practical experiments verified that the axis pose by our method had higher precision compared to the popular methods, and its position error reached around 0.8 mm with 90% confidence.

Moreover, our method, coupled with admittance control, was employed to achieve the shaft-in-hole assembly, where the fit clearance was about 1 mm. However, some further improvements should be made in this research: (i) the axis pose estimation in occlusion scenes needs to be taken into consideration, and (ii) the uncertainty of the axis pose under the robotic platform requires study.

## Figures and Tables

**Table 1:** The RMSE values of the angle and position in the axis pose estimation.

Method	${\mathrm{RMSE}}_{\phi }$ (${}^{\circ }$)	${\mathrm{RMSE}}_{d}$(mm)
RANSAC	0.4177	1.0892
ICP	0.3008	1.2153
PCA	0.3664	1.0267
Our method	0.0683	0.1727

**Table 2:** The RMSE values of the position in assembly.

Method	${\mathrm{RMSE}}_{d}$(mm)
RANSAC	6.0245
ICP	5.9782
PCA	5.5837
